# Long-term result of transcatheter arterial embolization for liver hemangioma

**DOI:** 10.1097/MD.0000000000009029

**Published:** 2017-12-08

**Authors:** Xiaolei Liu, Zhiying Yang, Haidong Tan, Jia Huang, Li Xu, Liguo Liu, Shuang Si, Yongliang Sun

**Affiliations:** Department of Hepatobiliary Surgery, China-Japan Friendship Hospital, Beijing, China.

**Keywords:** embolization, interventional radiology, liver hemangioma

## Abstract

Transcatheter arterial embolization (TAE) is a method for the treatment of liver hemangioma, but fewer studies reported the long-term result.

Retrospective study was conducted to liver hemangioma patients who received TAE. The inclusion criteria included the following: the period of follow-up was more than 5 years; and patients were followed up for less than 5 years, but received surgical treatment due to the enlargement of tumor or severe complications of TAE. The collected data included sex, age, size of the tumor, times of TAE, complications, period of follow-up, long-term result, and whether or not surgery was finally performed.

Fifty-five patients were included, and the average age was 43.1 ± 8.6 years. The average size of liver hemangioma was 9.0 ± 4.3 cm. Four patients (7.3%) had severe complications after TAE, including 2 cases of biloma which were cured by surgery. The tumor size was smaller or the same in 19 patients after 5 years follow-up, and the long-term effective rate was 35.8%. The size of tumor became larger in the other 34 patients (64.2%), and 29 patients (54.7%) received surgery finally. The long-term effective rate for patients with ≥10 cm tumor and <10 cm tumor were 12.5% and 45.9%, respectively, and the difference was significant (*P* = .019).

The long-term result of TAE for liver hemangioma was not satisfying, and the treatment had the risk of severe complication. For patients with asymptomatic liver hemangioma, TAE should not be conducted.

## 1 Introduction

Hemangiomas are the most common benign tumors of the liver with an estimated prevalence of 0.4% to 20%.^[1]^ Their pathogenesis is ill known, but many authors consider that they are vascular malformations or hamartomas of congenital origin that enlarge by ectasia, but not by hyperplasia or hypertrophy.^[2–4]^ Most hemangiomas are small and require no treatment because they exert no hazardous effects or damage on adjacent organs. Only giant liver hemangiomas that could cause symptoms require surgical interventions.^[5]^ Liver resection and hemangioma enucleation remain the most commonly used surgical procedures.^[6–9]^ With the development of interventional radiology, transcatheter arterial embolization (TAE) has become an effective treatment for hepatocellular carcinoma. It is also used as preliminary treatment for giant liver hemangioma before surgical procedure and emergency treatment for the rupture of giant liver hemangioma.^[10–15]^ Fewer studies reported the outcomes of TAE alone for liver hemangioma,^[16,17]^ and the long-term result was even rare. The aim of this study was to analyze the long-term result of liver hemangioma patients after the treatment of TAE.

## 2 Patients and methods

A search of the departmental database was conducted for liver hemangioma patients who visited our clinic and received the treatment of TAE. A 5-year period from January 2005 to January 2010 was reviewed. With the intention of shrinking the tumor or deterring the enlargement of tumor, the indication of TAE for liver hemangioma in that period was tumor larger than 5 cm with or without symptoms. The diagnosis of liver hemangioma was required to be confirmed by contrast-enhanced computed tomography (CT) or magnetic resonance imaging (MRI).

For all patients, TAE was performed using a standard procedure by the same doctor trained and experienced in this technique. Under local anesthesia, the right femoral artery was punctured with a 5-F arterial sheath using the Seldinger technique, and a 5-F hepatic artery catheter was pushed into the celiac artery, hepatic artery, and superior mesenteric artery via the arterial sheath for selective angiography. After confirmation of the feeding arteries and the location, size, and number of liver hemangiomas, a microcatheter was super-selectively placed into the feeding arteries, and a mixture of pingyangmycin and lipiodol was slowly injected through the catheter until the periphery of the hemangioma was completely surrounded.

After the treatment of TAE, patients were followed up annually with the examination of ultrasonography or CT scan. The inclusion criteria included the following: the period of follow-up was more than 5 years; and patients were followed up for less than 5 years, but received surgical treatment due to the enlargement of tumor or severe complications of TAE. The medical records of included patients were reviewed retrospectively. The collected data included sex, age, tumor size, symptom, times of TAE, complications, period of follow-up, long-term result, and whether or not surgery was finally performed. The indications for surgery were giant liver hemangioma (≥10 cm) with symptoms or Kasabach-Merritt syndrome. The size of the tumor alone was not considered an indication for operative therapy.

Informed consent was obtained from all patients regarding the diagnostic and therapeutic procedures. The study was approved by the Institutional Ethical Review Board of China-Japan Friendship Hospital.

### 2.1 Statistical analysis

The statistical analysis was performed using SPSS software (version 20.0, IBM Corp, Armonk, NY). Categorical variables are expressed as the number of cases, and continuous variables with normal distributions are expressed as the means ± standard deviation (SD). Categorical variables were compared using the chi-square or Fisher exact test. Student *t* test was applied for continuous variables with a normal distribution. Corrected *P* values <.05 were considered statistically significant.

## 3 Results

### 3.1 Demographics and complications of liver hemangioma patients with TAE

**Figure 1:**
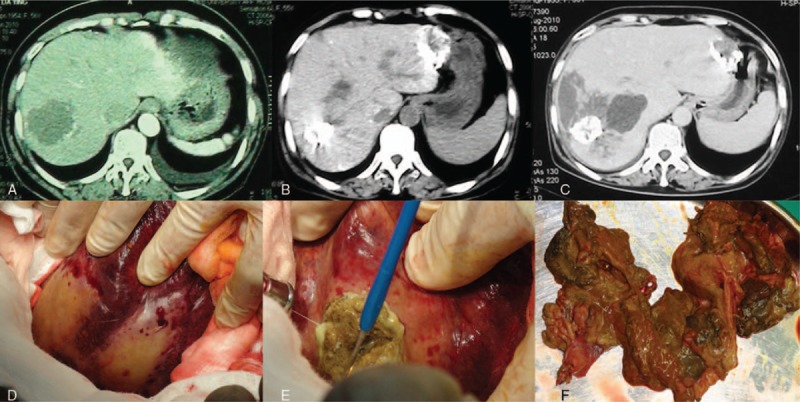
Biloma after the treatment of TAE. (A) CT scan before TAE; (B) CT scan after TAE; (C) CT scan after TAE, which showed the occurrence of biloma; (D, E) the photos during the surgery; (F) the specimen. CT = computed tomography, TAE = transcatheter arterial embolization.

**Figure 2:**
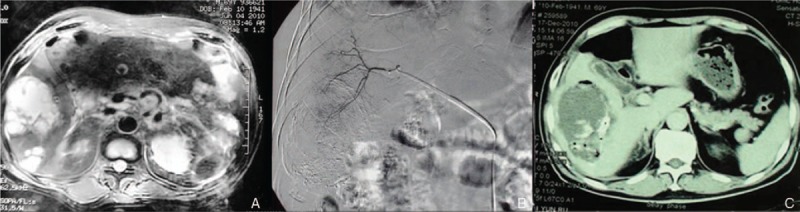
Liver abscess after the treatment of TAE. (A) MRI scan before TAE; (B) image during the treatment of TAE; (C) CT scan after TAE, which showed the occurrence of liver abscess. CT = computed tomography, MRI = magnetic resonance imaging, TAE = transcatheter arterial embolization.

In all, 63 liver hemangioma patients received TAE from January 2005 to January 2010. Eight patients were lost to follow-up and 55 patients were finally included (17 males and 38 females). The average age was 43.1 ± 8.6 years (range 27–71 years). The average size of liver hemangioma before the treatment of TAE was 9.0 ± 4.3 cm (range 5–29 cm). Thirty patients (30/55, 54.5%) had symptoms, including 20 cases (20/30, 66.7%) with abdominal distension, 8 cases (8/30, 26.7%) with abdominal pain, and 2 cases (2/30, 6.6%) with premature satiety after meal. Thirty-eight patients (38/55, 69.1%) received once TAE, 15 patients (15/55, 27.3%) received twice TAE, and 2 patients (2/55, 3.6%) received 3 times TAE. Four patients (4/55, 7.3%) had severe complications including 2 cases of biloma and 2 cases of liver abscess. One patient with biloma had infectious shock and received emergency surgery with the resection of segment VI and VII (Fig. 1). Another biloma patient received drainage firstly to treat the infection and then right hemihepatectomy was performed. Both liver abscess patients were cured with drainage (Fig. 2).

### 3.2 Long-term results of liver hemangioma patients with TAE

**Figure 3:**
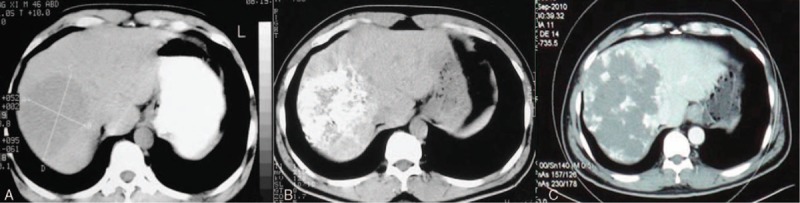
Change of liver hemangioma after TAE. (A) CT scan before TAE; (B) CT scan in 3 months after TAE, which showed the shrink of the tumor; (C) CT scan in 5 years after TAE, which showed the enlargement of the tumor. CT = computed tomography, TAE = transcatheter arterial embolization.

**Figure 4:**
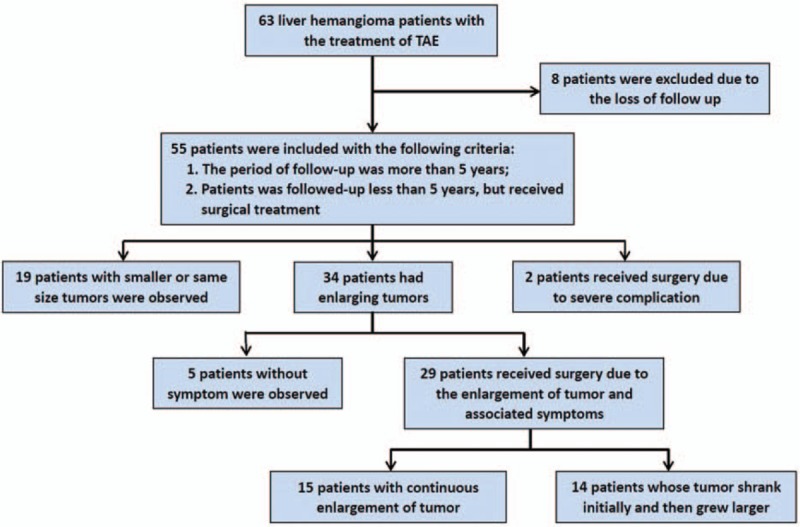
The long-term outcomes of liver hemangioma patients who received the treatment of transcatheter arterial embolization (TAE).

To calculate the long-term results, 2 patients who received surgery due to the complications after TAE were excluded. In 5 years after the treatment of TAE, 19 patients (19/53, 35.8%) had smaller or the same size of liver hemangioma, comparing with the status before TAE. Liver hemangioma of the other 34 patients (34/53, 64.2%) all grew larger. Twenty-nine patients finally received surgery due to the enlargement of tumor and the associated symptoms, including 14 patients whose tumor shrank initially and then grew larger (Fig. 3). Five patients with enlarging tumor were observed due to the inexistence of symptom (Fig. 4).

### 3.3 Comparison of demographics, complications, and long-term results between liver hemangioma patients with different sizes

According to the size of liver hemangioma, patients were divided into giant group (≥10 cm, 17 cases) and large group (<10 cm, 38 cases). There were no differences between giant and large groups in terms of sex (*P* = .322), age (*P* = .213), times of TAE (*P* = .097), and complication (*P* = .363) (Table 1). Giant group had higher rate of symptom (*P* = .000). Compared with large group, giant group had higher rate of cases with enlarging tumors (*P* = .019) and higher rate of patients in giant group received surgery finally (*P* = .000) (Table 1).

**Table 1:**
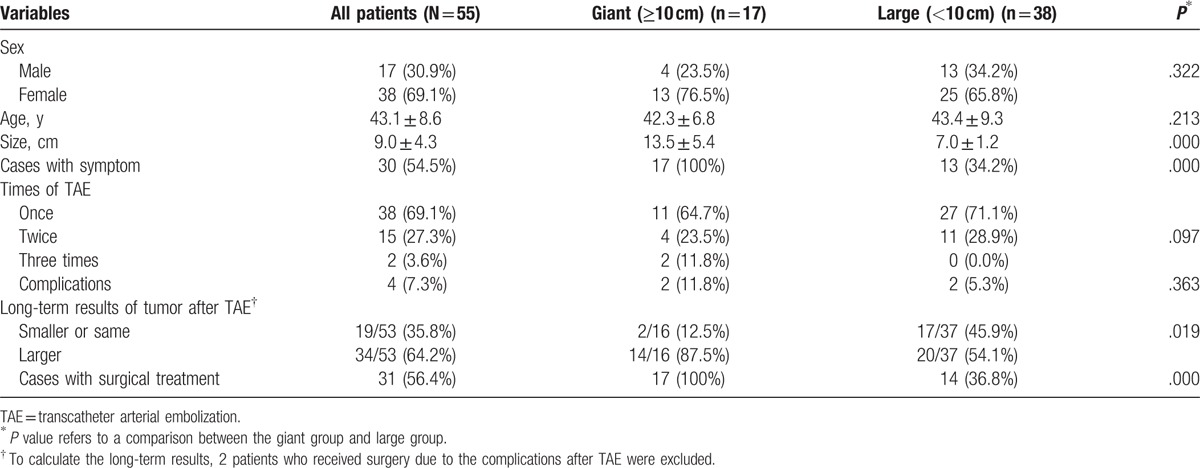
Demographics, complications and lone term results of liver hemangioma patients with TAE.

## 4 Discussion

Hemangiomas are the most common benign tumors of the liver with an estimated prevalence of 0.4% to 20%.^[1]^ These lesions consist of large vascular spaces lined by a single layer of endothelial cells that may grow slowly from birth. Most hemangiomas are small and require no treatment because they exert no hazardous effects or damage on adjacent organs. Only giant liver hemangiomas that could cause symptoms require surgical interventions. Most authors classify giant hemangiomas as those more than 5 cm,^[18–20]^ whereas a very small number of authors have defined giant hemangiomas as those more than 10 cm in size.^[21–23]^ Surgical resection remains to be the main treatment of liver hemangioma.^[24–27]^ Although the safety of surgical treatment increased recently, the risk of severe complications could not be totally avoided. With the improvement of interventional radiology and superselective catheterization techniques, TAE has become another option for the treatment of liver hemangioma. Compared with surgery, TAE was less risky, and previous reports showed that TAE could effectively shrink the tumor, which facilitated the surgical resection.^[10–15]^ TAE was also used alone to treat liver hemangioma, but only short-term results were reported.^[16,17]^ No study ever reported the long-term result of TAE for liver hemangioma. The result of this study showed that the long-term outcome of TAE was not satisfying. For most patients, TAE was incapable to stop the growth of liver hemangioma, especially for the patients with giant lesions (≥10 cm). Most of the patients received surgical treatment finally. The possible reason was that giant lesion had multiple nourishing vessels which made the total embolization impossible. Moreover, vasculogenesis may occur after the TAE and the tumor will get larger. Patients with 5 to 10 cm lesions had higher possibility that the tumor stayed stable or became smaller after the treatment of TAE. But the necessity of treatment was less, since <10-cm tumors had less possibility to cause symptoms.

Compared with surgery, TAE was less risky, but severe complications such as liver abscess and biloma could also occur. It has been reported in the treatment process of malignant liver tumors.^[27–30]^ The possible reason was the necrosis of bile duct. Due to the benign nature of liver hemangioma, the safety of the treatment should be primarily considered. Although the rate of complication was only 7.3%, it is still unacceptable due to the good prognosis of the disease itself. Moreover, the complication could be severe. Two patients with biloma in this study received surgical treatment because of acute cholangitis. Our primary intention of choosing TAE to treat liver hemangioma was to safely control the growth of liver hemangioma. But with the unsatisfying long-term result and the risk of severe complication, TAE was seldom conducted in our center for liver hemangioma patients recently. Surgical treatment should be the preferred treatment option for patients with giant liver hemangioma which caused symptom.

## 5 Conclusions

In summary, the long-term result of TAE for liver hemangioma was not satisfying and the treatment had the risk of severe complication. For patients with asymptomatic liver hemangioma, TAE should not be conducted. Surgical treatment should be the preferred treatment option for patients with giant liver hemangioma which caused symptom.

## Acknowledgments

The authors want to thank all of the associated staff of Radiology Department for assisting in the implementation of this study.
